# Segmentation of Substantia Nigra in Brain Parenchyma Sonographic Images Using Deep Learning

**DOI:** 10.3390/jimaging10010001

**Published:** 2023-12-19

**Authors:** Giansalvo Gusinu, Claudia Frau, Giuseppe A. Trunfio, Paolo Solla, Leonardo Antonio Sechi

**Affiliations:** 1 Department of Biomedical Sciences, University of Sassari, 07100 Sassari, Italy; g.gusinu@phd.uniss.it (G.G.); trunfio@uniss.it (G.A.T.); 2 Department of Medicine, Surgery and Pharmacy, University of Sassari, Viale San Pietro 8, 07100 Sassari, Italy; claudia.frau@aouss.it (C.F.); psolla@uniss.it (P.S.)

**Keywords:** image segmentation, deep learning, neuroimaging, parkinson, ultrasound, brain parenchyma sonography

## Abstract

Currently, Parkinson’s Disease (PD) is diagnosed primarily based on symptoms by experts clinicians. Neuroimaging exams represent an important tool to confirm the clinical diagnosis. Among them, Brain Parenchyma Sonography (BPS) is used to evaluate the hyperechogenicity of Substantia Nigra (SN), found in more than 90% of PD patients. In this article, we exploit a new dataset of BPS images to investigate an automatic segmentation approach for SN that can increase the accuracy of the exam and its practicability in clinical routine. This study achieves state-of-the-art performance in SN segmentation of BPS images. Indeed, it is found that the modified U-Net network scores a Dice coefficient of 0.859 ± 0.037. The results presented in this study demonstrate the feasibility and usefulness of SN automatic segmentation in BPS medical images, to the point that this study can be considered as the first stage of the development of an end-to-end CAD (Computer Aided Detection) system. Furthermore, the used dataset, which will be further enriched in the future, has proven to be very effective in supporting the training of CNNs and may pave the way for future studies in the field of CAD applied to PD.

## 1. Introduction

**Figure 1:**
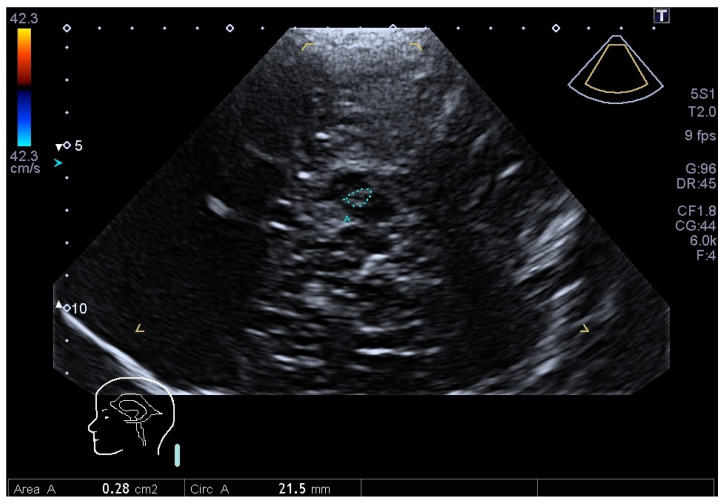
Transcranial sonography of the mid-brain in a PD patient through the left temporal acoustic bone window. The hypsilateral hyperechnogenicity of the SN within the butterfly-shaped mid-brain is encircled by a segmented blue line traced by the neurologist. The image is anonymized, and it will be cropped (cut) before entering the neural network in order to reduce the pixel imbalance between SN and background.

Parkinson’s disease (PD) is the second most common progressive neurodegenerative disease whose typical pathological hallmark is the loss of dopaminergic neurons of the Substantia Nigra (SN) of the mid-brain [1] and the deposition of alfa-synuclein in neurons [1]. The diagnosis of PD is typically clinical and is based on cardinal symptoms, including bradykinesia, rigidity, resting tremor and postural instability in later phases of the disease [2]. Despite the relevant expertise of the neurologist, clinical diagnosis is often challenging. Mostly in the early stage of the disease, PD may be confused with other disorders (essential tremor (ET), secondary and atypical parkinsonisms), which have different prognosis and management [3]. The gold standard is represented by neuropathological examination [4]. Several methods have been developed to help PD diagnosis. Important methods consist of functional imaging with Positron Emission Tomography (PET) or Single Photon Emission Computer Tomography (SPECT) [5]. These examinations use different presynaptic tracers to visualize the nigrostriatal system, and a presynaptic dopaminergic deficit, particularly if asymmetric, indicates idiopathic PD. Their use is limited because of the high costs and invasiveness. Furthermore, they may differentiate between PD and ET, but not between the former and PD mimics like atypical parkinsonisms, i.e., Multiple System Atrophy (MSA), Progressive Sopranuclear Palsy (PSP). Structural Magnetic Resonance Imaging (MRI) is generally normal in PD, while it may show some specific disease abnormalities of MSA or PSP, but only in later stages of the disease. Brain Parenchyma Sonography (BPS), using a transtemporal acoustic window (Figure 1), allows for the possibility of identifying SN hyperechogenicity, which is present in more than 90% of PD patients [6].

George Becker identified for the first time this echofeature, which was not seen before with other neuroimaging techniques [7]. BPS has thus become a reliable and valuable tool for the diagnosis of PD with a great specificity (82.4%), sensitivity (90.75%) and high predictive value for PD diagnosis (92.9%) [8]. When ultrasound waves propagate through tissue and encounter interfaces between two types of tissue, some of the sound waves will be reflected back. This reflected sound is defined the “echo" signal. The echo production is dependent upon acoustic impedance, a property of the tissue, as a result of its density and the propagation velocity of sound waves through that tissue. The causes of the echogenic modifications of SN are not well understood. It has been associated with increased SN iron content [9] and modifications in iron-binding proteins like decreases in neuromelanin content in SN [10]. It is a stable marker that does not correlate with severity or disease progression [11]. An important concept is that this echofeature is not directly correlated with the progressive loss of SN neurons, but it may be considered a marker of a certain vulnerability of the nigrostriatal system in PD patients, which could anticipate the onset of motor symptoms [12].

Compared to the other technologies mentioned above, BPS is a broadly available, quick, inexpensive and non-invasive method. However, its main limitations are represented by the necessity of an adequate temporal acoustic bone window and by the dependency on the skill of the operator. For these reasons, and also in view of the enormous progress made in recent years by Deep Learning (DL) in the field of computer vision [13], the present study aims to develop a tool to support the analysis of BPS images in such a way as to mitigate the most serious limitation of the technology in question when applied to the diagnosis of PD, namely the dependence of the method’s robustness on the availability of operators with particular skills. To this end, we created a new dataset of BPS images, which will be further extended in the future, in order to investigate the feasibility of automatic SN segmentation using Convolutional Neural Networks (CNN) [14]. The article reports a comparison between two of the most widely used and promising CNN architectures typically used for automatic segmentation, namely U-Net [15,16] and DeepLabV3+ [17], providing a methodology for effective training and indications of the expected accuracy, which, in spite of the relatively small size of the dataset available to date, proved quite satisfactory. As explained in the next section, results on the achievable accuracy of SN segmentation in BPS images by means of modern DL-based approaches are not frequent in the literature.

The Substantia Nigra is found in the mid-brain area. Image segmentation means to assign to each voxel/pixel of the image the status of true if it contains the PD indicator (hyperechogenicity of Substantia Nigra), or false if to the contrary. Several previous studies have tried to segment only the mid-brain. Very few studies have focused on the segmentation of the SN. In relation to the latter studies, our study compares two deep network architectures and shows higher performance (i.e., dice value).

The remainder of the article is organized as follows. In the next section, we discuss the state-of-the-art concerning the specific application of SN automatic segmentation. In Section 3, we give the details about the adopted methodology. Section 4 illustrates the outcomes of the algorithms under comparison and a detailed discussion follows in the subsequent Section 5. Finally, Section 6 concludes the article with some consideration on the achieved results and on the planned future work.

## 2. Related Work

After recognizing that Ultra Sound (US) technology can be valuable for the diagnosis of PD [12], several attempts have been made towards automatizing the analysis of transcranial sonography images. The first attempts were based on heuristic semi-automated methods. For example, in [18], after manually segmenting the interested area, the authors applied first a mask to highlight the SN and then, according to heuristic rules, some filters (i.e., selection of the largest object, dilation, mask, closing) to obtain the final SN segmentation. However, such an approach was still based on an initial manual segmentation and then strongly dependent on the skills of the operator. In [19], a complex pre-processing procedure was presented to improve the results of a modified active contour (AC) segmentation algorithm [20,21]. The latter iteratively fits an initially provided parametric curve (i.e., spline) to the target object (mid-brain) boundaries in the best manner possible. According to the presented results, the average overlap between regions obtained automatically and manually was 73.10±7.45 . However, the success of such a procedure strongly depends on the proper placement of the initial contour. Later, several studies that appeared in the literature were devoted to detecting or segmenting in US images only the mid-brain, almost always missing the final stage of automatically segmenting the SN region, i.e., [22,23,24,25,26]. For example, in [22] the authors presented a semi-automatic mid-brain segmentation method from 3D TC-US. The technique was based on the application of shape models [27]. However, besides leaving to a subsequent manual stage the SN segmentation, the approach did not perform well for uncommon mid-brain shapes and was based on a small dataset (11 patients + 11 healthy controls). A study addressing the detection of SN in 3D TCS is described in [28], where the authors devised an algorithm combining prior knowledge and a classifier based on Random Forest that, starting from a set of labeled data, was able to provide for each voxel of an unseen image the probability of belonging to the SN region. However, the study was based on only 22 patients, and the reported F measure of 0.519±0.148 was not particularly satisfactory. The authors of [23] presented a fully automated method for segmenting several anatomical structures (prostate, left ventricule, mid-brain) in 3D ultrasound images with a Hough Forest-based framework, obtaining a DICE coefficient of 0.85±0.03 for the mid-brain. However, again the used dataset was very small (12 ultrasound volumes), and the work did not address SN segmentation. Moreover, it is not clear the percentage of data used for testing and nor, therefore, the actual reliability of the approach. The study described in [24] adopted a multi-domain regularized deep learning method for anatomical structure detection and segmentation in ultrasound images. The method was based on fully convolutional networks by leveraging the transfer learning approach but did not address explicitly the SN segmentation issue. Another heuristic algorithm for helping in the detection of the mid-brain area in US images was presented in [25]. The proposed approach assumed that the shape of the mid-brain is almost constant, being the differences only in width, length and orientation. According to the results, the algorithm performed relatively poorly when the mid-brain shape differed from the average shape and, again, it did not address SN segmentation. The authors of [26] used a statistical shape model algorithm based on 90 manually delineated contours to segment the mid-brain. Then, they applied a pixel-level classifier-based segmentation strategy for the extraction of the SN region. The study was based on a dataset of 191 individuals, and the reported DICE coefficient was in between 0.64 and 0.66 for the SN, which is much lower than the values obtained in the present study. Some other recent studies presented in the literature were based on the segmentation of the brain structure, but not specifically for the mid-brain region. For example, in [29], a fully convolutional deep segmentation architecture was used in combination with pre-training on simulated data for the segmentation on 3D-ultrasound images. The work proved that pre-training the network can lead to better generalization.

Also worth mentioning is a relatively recent and comprehensive review of DL techniques applied in the field of medical image analysis presented in [30]. A more specific review was published about neural network applications for supporting the detection of PD in [31]. According to the latter study, that analyzed 91 full-text peer-reviewed studies, and most of the datasets were based on bio-metric data (i.e., voice, EEG, EMG) and some were based on medical imaging (MRI, CT, PET, DaTscan). However, out of the 91 article isolated by the authors of the study, no one used US medical images or calculated the SN area to help detect PD. Furthermore, most of the studies makes use of detection and/or classification, whilst only a small fraction of them were based on segmentation techniques.

In the literature, one can find several studies based on medical images segmentation—for example, US breast image segmentation [32], thyroid nodule segmentation [33], cardiac image segmentation [34], liver segmentation [35], kidney segmentation [36].

In [32], the authors improved US breast image segmentation by designing a new network model based on U-Net. They added a bidirectional attention guidance network (BAGNet) and a refinement residual network (RFNEt). In particular, the first U-Net is used to generate a set of low-level and high-level features. The BAGNet is used to capture the context between low-level and high-level features. Finally the residual refinement network is applied to learn the difference between feature maps and ground-truth masks. In future test, it would be interesting to apply this network model to our dataset and see how they would perform.

In [33], the authors address the automatic thyroid nodule segmentation. They proposed a “Super-resolution reconstruction” method for cleaning the US images prior to passing them to the following stages. The following stages are quite complex and require an N-shape network (consisting of several ASPP blocks), a multi-scale input layer, attention module and a PAC module (constructed to accurately segment the thyroid module). Several limitation were highlighted by the authors; however, it would be interesting to merge some of our findings.

In [34], the authors propose a new framework to improve three-dimensional (3D) cross-modality cardiac image segmentation, which they say is critical for cardiac disease diagnosis and treatment. Their causal knowledge fusion (CKF) framework focuses on the anatomical factor and discards the modality factor because of the fact that the anatomical factor is the causal invariant representation that transfers between different modalities. After testing their framework on the cardiac images of 503 MR patients and 518 CT patients, they claim that their framework is effective (Dice > 0.949) and superior to eighteen state-of-the-art segmentation methods.

The problem of kidney segmentation with a limited dataset size (i.e., MR scan images from only a few subjects) has been addressed in [36]. The authors used the CHAOS public dataset plus a private dataset. They tested two network models: single U-Net with a backbone of ResNet34 and two cascaded U-Net. The second U-Net was a slightly modified U-Net with four channels composed of three slices and one mask for the third slice. The two network were trained independently with 1, 3 or 6 subjects. They claim that the cascaded U-Net performs better than the single U-Net when using only an MR scan image from three patients (Dice value 0.893 vs. 0.864).

Overall, based on the literature review outlined above, despite the enormous progress in the field of DL-based segmentation and the wide availability of the corresponding algorithms, the problem of ascertaining the achievable accuracy of automatic SN segmentation in US images has not been sufficiently addressed. Such a gap, probably due to the limited availability of the necessary datasets, will be addressed in the remainder of this article.

## 3. Materials and Methods

We retrospectively studied 23 patients that were evaluated in the Movement Disorders Centre of the Neurological Clinic of Sassari between June 2020 until June 2022.

As summarized in Table 1, the participants of the trial were 23 PD positive patients and 8 healthy controls, 18 males and 13 females, aged from 52 to 84. All patients fulfilled all criteria for the diagnosis of PD [2]. Furthermore, the 8 healthy controls were not under investigation or treatment for any neurodegenerative disease. The study was approved by the local ethics committee (Prot. PG/2023/7846) and was performed according to the Declaration of Helsinki. Informed consent was obtained by all participants.

Inspired by NiftyNet [37], a small framework based on Python and TensorFlow has been developed. This is useful to easily implement and test various network models, test different datasets (see Table 2) and tune the hyper-parameters in a efficient and scalable way. The framework is available for download as explained in the Data Availability Statement.

### 3.1. BPS Imaging

BPS imaging was performed using an ultrasound machine, Toshiba Aplio 500, equipped with a phased array sector probe (2–2.5 Mhz). The main parameters of ultrasonic scanning were those recommended in the literature [43]: image depth: 14–16 cm, dynamic range 45–55 dB with gain compensation and image brightness adapted manually as needed. All US scans were performed by a neurologist trained to vascular and parenchymal sonography (C.F.). During the examinations, the subjects were sitting on supine position. Scans were made in a mid-brain axial scanning plane in B-mode using bilateral temporal bone acoustic window. The transducer was placed at the posterior or middle temporal bone, parallel to the orbito-meatal line. In this position the butterfly-shaped mid-brain was visualized as clearly as possible. In the mesencephalic plane, the area of the SN was measured (in cm^2^), after manual encircling of the entire circumference of the echogenic SN area (see Figure 1). The normative threshold of the SN area calculated in our laboratory were those accepted by guidelines: marked hyperechogenicity Area > 0.25 cm^2^; moderate hyperechogenicity 0.20 cm^2^ < Area < 0.25 cm^2^; normal echogenicity Area < 0.20 cm^2^ [44].

### 3.2. Selection of CNN Models

A certain number of tasks were necessary before the final stage of training in order to identify the best candidate network and to prepare the images for our Parkinson dataset. Two networks were chosen for this study: U-Net and DeepLabV3+.

**Figure 2:**
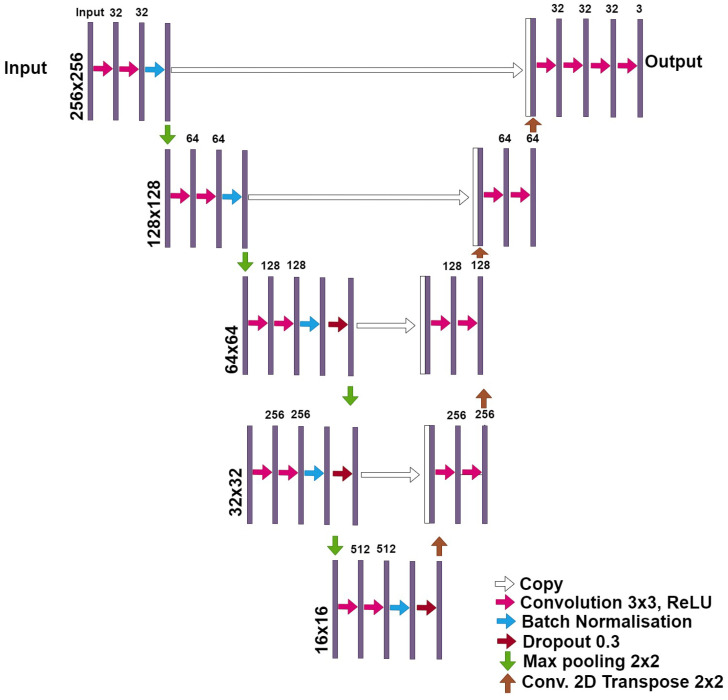
The modified U-Net network architecture used in this study. Each box corresponds to a multi-channel feature map. On top of each box is written the number of channels. The x-y size is provided at the lower left edge of the box. White boxes represent copied feature maps. The arrows denote the operations. This architecture differs from the original U-net model because of input image size, Dropout layers and Batch Normalization layers.

The U-Net model is widely used in the medical imaging research field, where it has become the de-facto segmentation standard because it has proven to be reliable and efficient [45,46]) and it has been deeply studied in various articles (e.g., in [47,48]). Several versions of this model are publicly available, with subtle differences in the sequence of layers, activation function, and other characteristics. However, they usually maintain the original “auto-encoder” structure with the “skip” connections. Figure 2 shows the modified U-net architecture used for this research.

The architecture of the modified-U-net network differs from the original U-net model because of several factors. The input image size of plain U-net is 572 × 572 pixels, the U-Net network converts a gray scale input image of size 572 × 572 × 1 into a binary segmented output map of size 388 × 388 × 2. We can notice that the output size is smaller than the input size because no padding is being used. Instead, our modified-U-net can receive, as input, color images (RGB coded) of different sizes (from 200 × 200 × 3 px to 300 × 300 × 3 px). Another difference between the two networks are the dropout layers present in the third and four layers in the modified U-net. A dropout layer randomly sets input units to 0 with a frequency of rate at each step during training time, which helps prevent overfitting. The third and last difference between the two architectures is the Batch Normalization function present in all layers of the modified-U-net. Batch normalization is a method used to make the training of artificial neural networks faster and more stable through the normalization of the layers’ inputs by re-centering and re-scaling. For more details about batch normalisation and dropout layers, see findings in [49].

**Figure 3:**
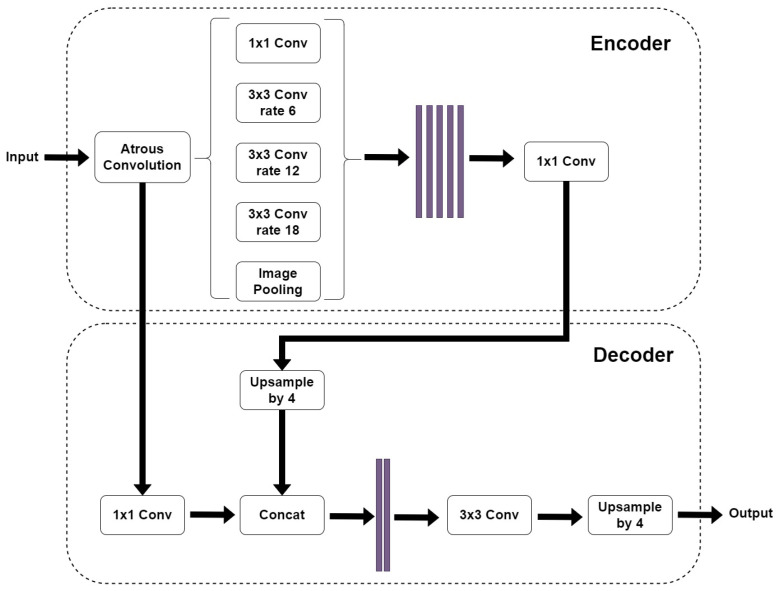
The adopted DeepLabV3+ network architecture [17]. The input image enters an “Atrous Convolution” block. The resulting object is sent to two different paths: the “spatial pyramid pooling” block in the encoder and the 1 × 1 convolution block in the decoder. The encoder path is then up-sampled by four and concatenated with the previous path. The resulting image is then convoluted and up-sampled to give the output image.

The DeepLabV3+ model, Figure 3, is an improvement of the previous DeepLabV3 model [50]. The older model uses the concept of “spatial pyramid pooling” which captures rich contextual information by pooling features at different resolutions. Moreover, the DeepLabV3+ model adds to this technique the “encoder-decoder” structure, which is able to obtain sharper object boundaries. Furthermore, the DeepLabV3+ introduces adjustments in order to perform atrous convolution on limited hardware [17]. The DeepLabV3+ model that we used is composed of a DeepLabV3 encoder followed by an Xception backbone as a decoder because during our preliminary tests, it proved to be more efficient than the MobileNetV2 backbone.

We downloaded several publicly available implementations of both models and tested them with a Python script that was written specifically to compare several models and to be scalable. Several hyper-parameters’ configuration were tested. Finally, we kept only one implementation of each model and a few configurations [51,52]. During the integration phase, in order to test the software, improve the script and build up reliability, we tested the whole framework on some non-medical datasets, such as “The Oxford-IIIT Pet Dataset” [41], “The ImageNet Dataset” [38], “The PASCAL Visual Object Classes Challenge (VOC)” [39], “The Cityscapes Dataset” [40].

### 3.3. Data Preparation

**Figure 4:**
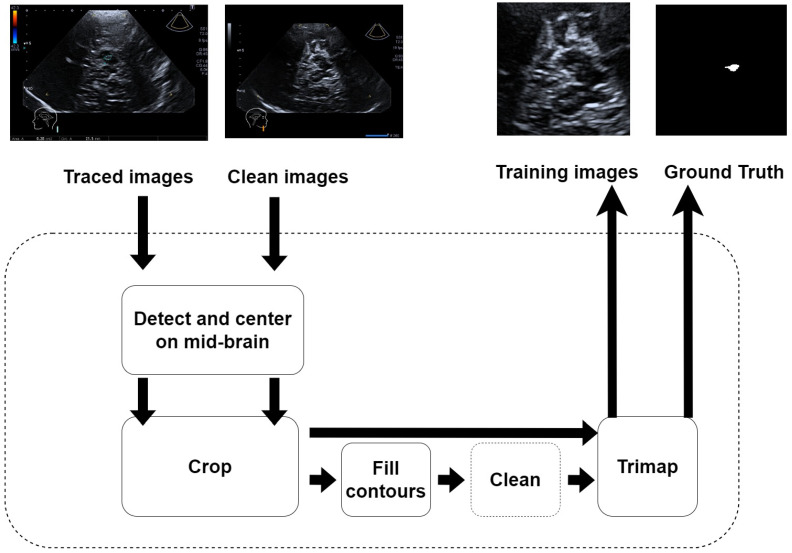
Pre-processing flow for creating the Parkinson’s dataset. All staging were performed automatically with a specific Python script, except for the cleaning stage, which was performed “manually” with the software GIMP 2.10.32. The inputs are the US image of the individual and the same images traced by the neurologist. In the first stage, they are anonymized to remove all sensible data of the individual. Then, they are centered and cut (cropped) in order to reduce the imbalance between “useful” pixels and background pixels, and to highlight the mid-brain region. The “clean image” becomes the training image, whilst the traced image becomes the “Ground Truth” after being filled and cleaned.

Apart from preparing and integrating the network models into the framework, the other task was to prepare the images and to prepare our “Parkinson” dataset. The neurologist took care of providing two images for each ultrasound scan: the first image was clear of any artificial annotation, and the second image was the same as the first plus a segmented non-continuous line reporting the contours of the SN as shown in Figure 1. A specific Python 3.8 software was developed to pre-process these images and get the final annotations with a continuous contour and the content filled with solid color. The process is depicted in Figure 4. It was semi automatic, as it required some “manual” refinement during the cleaning stage to obtain the ground truth trimap images.

This is surely a weak point in the whole procedure and should be improved in future investigations. All the images were taken in standard B-mode, or Doppler mode. The images were automatically cropped from the original size of 960×720 pixels (class imbalance 0.1% ) to the size of 300×300 pixels (class imbalance 1% ) by isolating their central part. This cropping operation tremendously improves the efficiency of the framework without affecting scalability, because the mid-brain area is usually found at this location [43]. Finally, two version of the Parkinson dataset were created: the first one where the ground truth has 2 classes (foreground, background) per pixel and the second one that has 3 classes (foreground, border, background) per pixel.

As summarized in Table 3, the dataset was split in three sets: training set (70%), validation set (10%), test set (20%). For each subject, two images were taken: one from the left side and the other from the right side. One image was not taken because the thickness of the temporal bone did not allow the neurologist to take the TCR image.

### 3.4. The Pre-Training Stage

Given the small size of our dataset, we adopted a fine-tuning approach. In short, the network was first trained in a dataset much larger than the available dataset, but with similar characteristics. Then, a final training on the target dataset aimed to refine the network’s weights to maximize its segmentation capacity for the specific application of SN segmentation. For the purpose of pre-training, we selected, after a series of tests with various candidate datasets (see Table 2), the “Kaggle Ultrasound Nerve Segmentation dataset” [42]. The latter is a collection of ∼5600 B-mode ultrasound images (and annotations) of the neck. The dataset was made available for a contest where users were challenged to propose a network model that could identify nerve structures in a dataset of ultrasound images of the neck. Each images in the training dataset is annotated with two classes per pixel (foreground/background).

Here, several training were again made in order to find the hyper-parameters that maximize the segmentation. Each training lasted around 2/3 h on a machine with a single CPU Intel Xeon (Intel, Santa Clara, United States) at 2.27 GHz with 24 GiB and NVIDIA GPU (NVIDIA, Santa Clara, United States) K-40c at 0.745 GHz and 12 GiB. The best results were obtained with the U-Net model using dropping layers and DeepLabV3+ with Xception backbone. The weights from this training were saved in order to be used later with our actual Parkinson dataset. All the results were compared using the Dice Similarity coefficient (DICE in short), which can be expressed by the following formula:

(1) $$DICE=DSC=2*\frac{X\cap Y}{X\cup Y}=\frac{\sum_{i}x_{i}*y_{i}}{\sum_{i}x_{i}+\sum_{i}y_{i}}$$

The DICE coefficient ranges from 0 (no similarity at all) to 1 (perfect match) and is used to compare the ground truth (obtained from the experienced neurologist) to the network prediction.

### 3.5. The Training Stage

Eventually, the two selected networks were tested on the Parkinson dataset in three different configurations:

- from scratch;
- fine tuning after pre-training on Ultrasound Nerve dataset;
- transfer learning (from Ultrasound Nerve dataset) freezing encoder or decoder.

**Figure 5:**
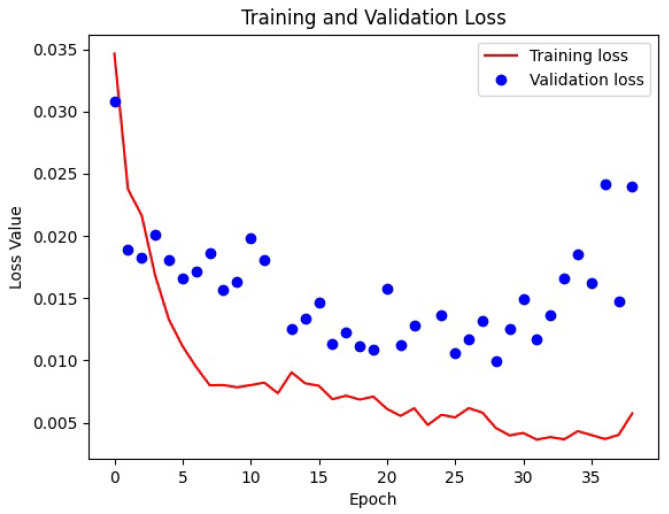
Plot of loss functions obtained during the training that later revealed to be the best one. The training finished after 37 epochs, in 3′42″, on a computer equipped with CPU Xeon @2.27 GHz/24 GiB and GPU K-40c/0.745 GHz/12 GiB. Early stopping was set to 10 epochs in order to reduce overfitting.

The transfer learning was accomplished by freezing the encoder or the decoder. In accordance with what was expected, the best result was obtained with weight initialization (from Kaggle Ultrasound Nerve dataset) and transfer learning (freezing the decoder). Loss functions in deep learning are used to measure how well a neural network model performs. The scope of the network is to minimize such function in the back-propagation algorithm. In our case, the SparseCategoricalCrossentropy function from the Tensorflow Keras library was used. An epoch refers to one complete pass of the entire training dataset through the learning algorithm. In other words, when all the data samples have been exposed to the neural network for learning patterns, one epoch is said to be completed. To improve the time of training of a network, in order to not waste time, it is possible to use an early stopping parameter. This tells the network to stop training if there is no improvement for a certain number of epochs. The loss functions of the best training are visible in Figure 5.

Several trainings were conducted on both selected network models to test for other hyper-parameter changes: augmentation, learning rate, image size, transfer learning, number of classes per pixel in the ground truth. The augmentation technique was limited to horizontal flip because other augmentation techniques (vertical flip, rotation, zooming) showed no improvements. The learning rate was tested for 1.00 × 10^−2^, 1.00 × 10^−3^ and 1.00 × 10^−4^ values and the best result were obtained with a value of 1.00 × 10^−3^. Several training sets were repeated to identify the best image size (128, 248, 256, 300 pixels). The U-Net model showed the best results for bigger values, the best being 256 due the network limits. Even the DeepLabV3+ model showed the best result with 256 pixels, but showed no specific trend. The average time of training for the U-Net model was around 10 min, whilst the average time of training for DeepLabV3+ was around 20 min on the hardware cited in Figure 5.

## 4. Results

During this study, many hours were spent tuning the framework’s parameters and training the network models. Some intuitions proved to be wrong, with the DICE going lower than 0.6, and they have been discarded. For the sake of completeness, we report here some of these experiments before giving the final results.

To overcome the small size of the dataset, we employed some well known augmentation technique (A data augmentation technique consists of generating some random variations in the dataset in order to increment the difference between samples and thus the information received by the network during the training stage): random horizontal flip, random translation, random rotation. As we can see in Table 4, in some cases the network training did not converge (Horizontal Flip + Translation for both networks, Flip+Rotation for DeepLabV3+). This effect may be caused by too big a displacement of the SN inside the sample image, or it may be that the augmentation created too many different situations to learn efficiently. In future experiments, it would be useful to zoom out the sample images or to reduce the maximum angle/offset.

We also explored the possibility of pre-training the networks. In fact, one of the U-Net implementations that we used came with the possibility of loading weights from ImageNet [38]. We slightly modified the code to allow the freezing (The weights in the frozen layers do not get updated during the training process) of some layers. This technique is called “Transfer Learning” because the idea is to pass the learning obtained on a given dataset to another dataset. In particular, we initialized the U-Net model with the pre-trained weights and froze the encoder once (DICE =0.5395 ), then the decoder (DICE =0.5478 ). As the reader can notice, in both cases the results are quite poor.

Then, we compared the two available sub-models of DeepLabV3+ (Xception and MobileNetV2) with pretrained weights. The results are shown in Table 5. Here again, the DICE values obtained are always lower than a sufficient threshold.

**Figure 6:**
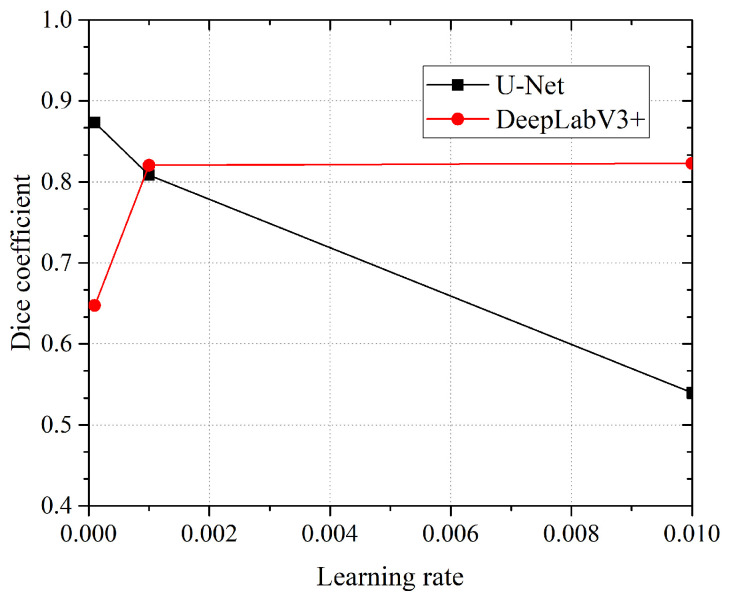
Dice coefficient obtained with three different learning rates (0.0001, 0.001, 0.01) by the two network models. The best performance is obtained by modified U-Net (DICE = 0.8732@0.0001). The network DeepLabV3+ shows best performance at learning rate 0.01 (DICE = 0.8229).

Another parameter that we decide to test for was the “learning rate”. The learning rate deals with the derivative of the loss function. In simple words, this parameter controls the speed at which the network learns (hopefully converging to a minimum). This is useful when using pre-initialized weights and/or transfer learning. Three different rates were analyzed in the study for the two network models: 0.01, 0.001, 0.0001. As we can see in Figure 6, the U-Net network obtains better DICE coefficient values for a higher learning rate (DICE is 0.8732@0.001), whilst the DeepLabV3+/Xception network obtains higher DICE coefficient values for a lower learning rate (DICE is 0.8229@0.01). For all tests, the networks were initialized with the “Kaggle Ultrasound Nerve” pretrained weights.

**Figure 7:**
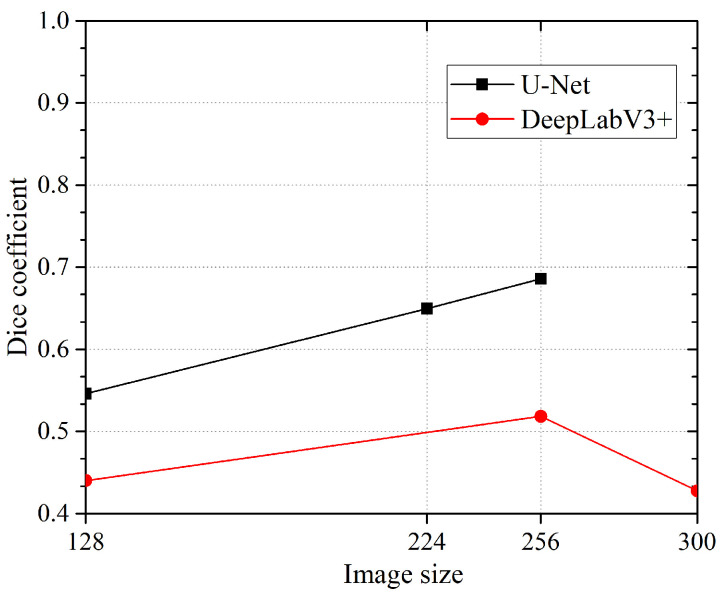
Dice coefficient obtained with different image sizes. Modified U-Net always outperforms DeepLabV3+. The best performance is obtained by modified U-Net (DICE = 0.6859@256 × 256 pixels), which shows better performance at higher image size. The network DeepLabV3+ also shows best performance at 256 × 256 pixels (DICE = 0.5184) but does not highlight a clear trend.

We thought that it was interesting to check what happens when the image size given to the network changes. In this case (see Figure 7), we get an interesting trend for U-Net where the DICE value is proportional to the image size (DICE = 0.6859@256 × 256 pixels). Unfortunately, the implementation of the U-Net we employed does not allow us to use images bigger than that. The DeepLabV3+, on the contrary, showed an unexpected fall from 256 to 300 pixels (max DICE is 0.5184@256 × 256 pixel).

Finally, as the reader can see in Table 6, the best result was achieved by the U-Net model with transfer learning (DICE 0.859±0.037 ), followed by the U-Net model from scratch (DICE 0.819±0.110 ), then the U-Net with fine tuning (DICE 0.806±0.152 ). However, the last two are very close. DeepLabV3+ was not tested with transfer learning because the model did not support it. It could be interesting to modify the code in order to overcome this limitation. The best performance by DeepLabV3+ was obtained with fine tuning (DICE 0.687±0.143 ), followed by “from scratch” configuration (DICE 0.540±0.0 ). The best configuration used the following parameters: two-class annotation dataset, image size is=256×256 pixels, learning rate $lr=10^{-3}$ , drop rate dr=0.3 . All training was conducted with a 5-fold validation method technique instead of the usual tenfold validation method to reach a trade-off between the statistical validation and the low number of samples in the dataset. This could be improved with a higher number of samples.

**Figure 8:**
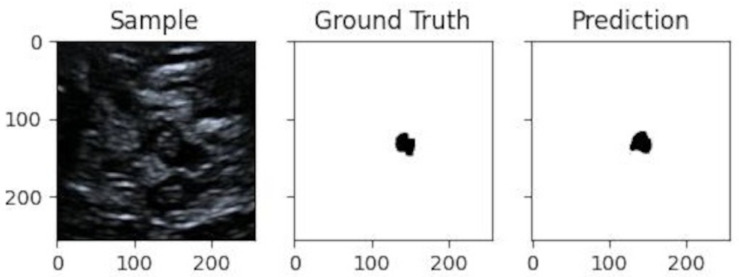
Qualitative result for one sample image from the test-set. It shows, from left to right: sample image, ground truth obtained from the human expert through some pre-processing, and prediction by the network.

**Figure 9:**
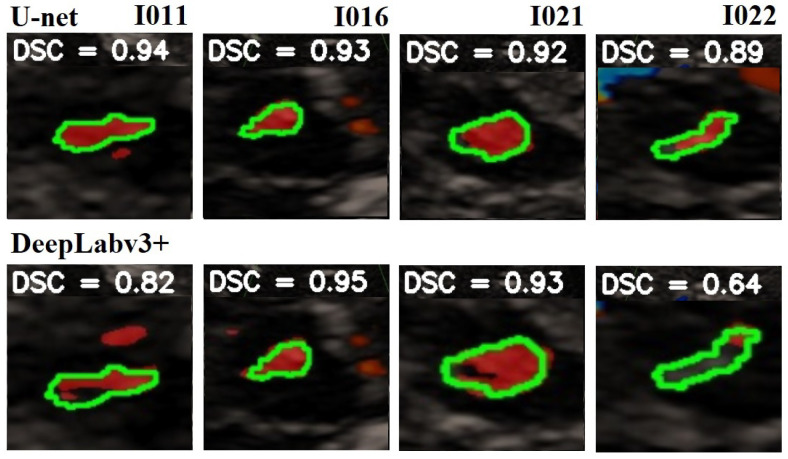
Comparison of the SN segmentation achieved by the two network models. The ground truth is highlighted in green, the prediction is in red. In the background is visible the typical butterfly-shaped mid-brain area (in black). The first row shows the results of modified U-Net, and the lower row shows the results of DeepLabV3+. The red areas out of the green lines indicate false positive. The black areas inside the green lines indicate false negative. For a better observation, the images are out of range.

**Figure 10:**
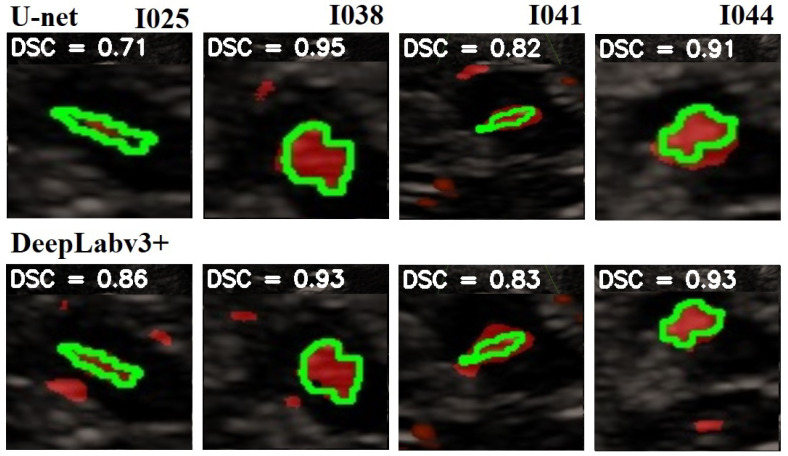
Comparison of the SN segmentation achieved by the two network models. The ground truth is highlighted in green, the prediction is in red. In the background is visible the typical butterfly-shaped mid-brain area (in black). The first row shows the results of the modified U-Net, and the lower row shows the results of DeepLabV3+. The red areas out of the green lines indicate false positive. The black areas inside the green lines indicate false negative. For a better observation, the images are out of range.

**Figure 11:**
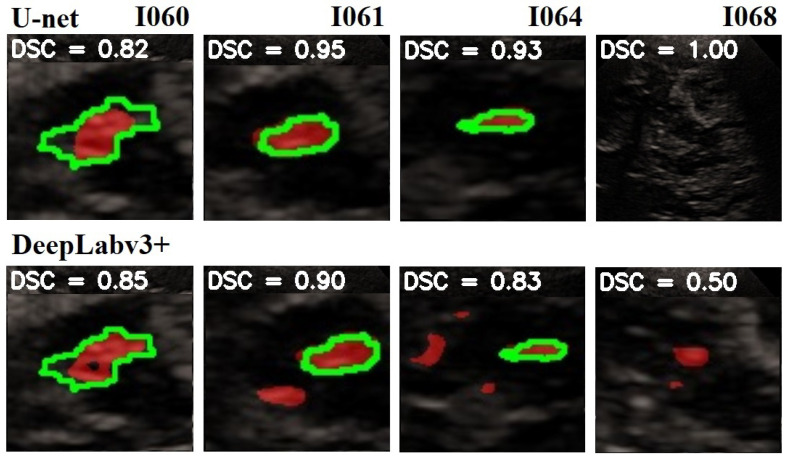
Comparison of the SN segmentation achieved by the two network models. The ground truth is highlighted in green, and the prediction is in red. In the background is visible the typical butterfly-shaped mid-brain area (in black). The first row shows the results of modified U-Net, and the lower row shows the results of DeepLabV3+. The red areas out of the green lines indicate false positive. The black areas inside the green lines indicate false negative. For a better observation, the images are out of range.

In Figure 8 is given a black and white example where one can compare the prediction from the network with the ground truth. In Figure 9, Figure 10 and Figure 11, we can see some qualitative and quantitative results obtained by the two network models on all samples of the test set (in a single execution). As the reader can see, in this particular execution, U-Net performed better than DeepLabV3+ on samples I011, I022, I038, I061, I064, I068, while DeepLabv3+ performed better on samples I016, I021, I025, I041, I044, I060. Note that the last sample, I068, is related to a healthy control individual, and therefore, it does not show any evidence of SN (the green contour is absent).

## 5. Discussion

We aimed to assess the diagnostic ability of PD diagnosis using BPS thanks to an automatic system. A lot of progress on BPS quality has been made, but nowadays, a very important limit of sonographic B-mode evaluation is still the dependence on the sonographer’s experience and skill. The use of software allows the possibility of partially eliminating it.

Several limitations of our study should be mentioned. The small dataset size and the low resolution of each sample image (compared to MRI and CT scans) are examples. In fact, the quality of BPS images is influenced by the quality of the ultrasound machine, sonographer’s manual measurements and sonography system setting. Changes in settings may influence the image brightness and could lead to a bias. A very useful method could be the MR imaging-TCS fusion imaging with virtual navigation technology. It allows simultaneous real-time TCS exam and MR images with more exact identifications of cerebral structures. The association of TCS-MR fusion with U-Net analysis could be a very important diagnostic tool for in diagnosis of PD.

## 6. Conclusions

In this article, using two different Convolutional Neural Network architectures (modified U-Net and DeepLabV3+), we analyzed and demonstrated the feasibility of SN segmentation in BPS 2D US medical images. As we used (almost) standard CNN network models, we believe that our approach is much simpler than quite complex solutions found in the literature and outlined in Section 2. Moreover, we payed attention to the scalability of the approach, avoiding overfitting of the models and achieving a good trade-off between complexity and performance, paving the way to a real-time system for PD detection.

In particular, the modified U-Net model pretrained on a public US dataset combined with transfer learning and fine tuning (dropping layers, frozen decoder) reached a satisfactory DICE mean value of 0.859±0.037 .

Further studies would be necessary in order to evaluate the size of the SN area and to integrate the software into medical devices. This would eventually lead to completing the process of developing an end-to-end CAD system for PD detection.

Firstly, when preparing the dataset, the region extracted from the original images and its size could be dynamically selected to reduce class imbalance and improve the efficiency of our framework. For example, this could follow one of the methods mentioned in Section 2.

Secondly, the accuracy of the algorithms used in the present study could be improved by increasing the dataset size. Moreover, to generalize our findings, it would be interesting to include images taken from other US devices. In fact, the lack of large and general datasets is a common limitation in all medical informatics research.

Another interesting improvement for this study would be to transition to an unsupervised deep learning approach, as suggested by some authors for other pathologies, e.g., [53]. This could reduce the development time and the workload on physicians and software engineers required to prepare the data and build and validate the software.

## Figures and Tables

**Table 1:** Number of patients and healthy controls who participated in the trial. In total, there were 31 individuals (23 patients and 8 healthy controls), 18 males and 13 females.

	Male		Female		
	n.	%	n.	%	Total
Patients	14	60.9%	9	39.1%	23
Healthy Controls	4	50.0%	4	50.0%	8
Total	18	58.1%	13	41.9%	31

**Table 2:** Description of the datasets used in the study. The first four datasets are well known and are used as benchmarks for novel algorithms and neural networks. They are composed of “normal” images, and they have been used in this study to validate the software (developing, debugging, testing). The “Nerves Ultrasound” dataset is made of US images, and it has been used for the transfer learning technique in this study. The “Parkinson” dataset has been created by the authors and used in this study to show the feasibility of the segmentation of SN in US images.

Dataset	# of Images	Reference
ImageNet ILSVRC2016	20,000	[38]
PASCAL VOC	7000	[39]
Cityscapes	25,000	[40]
Oxford-IIIT PET	7300	[41]
Nerves Ultrasound	5600	[42]
Parkinson (our)	63	This article

**Table 3:** Number of images in training, validation and testing sets in our dataset. The dataset is composed of 61 images. Two US images were taken for each individual (one from the left side and one from the right side). For one individual, one image was not taken because the temporal acoustic bone was too thick for the US to work. The dataset was split in the following training/validation/test ratios (70%, 10%, 20%).

	# of Samples	%
Training	43	70%
Validation	6	10%
Testing	12	20%
Total	61	100%

**Table 4:** Dice value obtained with some augmentation technique. The best result (DICE = 0.819) was obtained by modified U-net with Horizontal Flip (swap left and right). The second best value (DICE = 0.6452) was obtained by U-net modified with Horizontal Flip plus Rotation. The third best value (DICE = 0.540) was obtained by DeepLabV3+ with Horizontal Flip. In all other combinations, the training failed to converge (N/A).

	U-Net	DeepLabV3+/Xception
Horizontal Flip	0.819	0.540
Horizontal Flip + Rotation	0.6452	N/A
Horizontal Flip + Translation	N/A	N/A

**Table 5:** Comparison of the two versions of DeepLabV3+ (Xception vs. MobileNetv2) with pretrained weights. Neither model reaches satisfactory performance (the DICE value is always below 0.6).

Network Model	PASCAL VOC	Cityscapes	None
DeepLabV3+/MobileNetv2	0.5406 ± 0.0156	0.5436 ± 0.0041	0.5436 ± 0.0041
DeepLabV3+/Xception	0.4815 ± 0.1441	0.5437 ± 0.0041	0.5400 ± 0.0

**Table 6:** Summary of the performance achieved by the two network models (U-Net and DeepLabV3+) on the 2-class dataset, using three different training strategies: A. from scratch, B. fine tuning after pre-training on Ultrasound Nerve dataset, D. transfer learning from Ultrasound Nerve dataset. The values indicated are the Dice mean and standard deviation after 5-fold trainings.

Network	A. From Scratch		B. Fine Tuning		C. Transfer Learning	
	Mean	SD	Mean	SD	Mean	SD
U-Net	0.819	0.11	0.806	0.152	0.859	0.037
DeepLabV3+	0.540	0.0	0.687	0.143	N/A	N/A

## Data Availability

The software developed for this study is available online at https://github.com/giansalvo/preprocess (accessed on 1 September 2023) and https://github.com/giansalvo/segmentation (accessed on 1 September 2023). The data that support the findings of this study are available from the corresponding author, L.A.S., upon reasonable request.

## Abbreviations

The following abbreviations are used in this manuscript:

BPS	Brain Parenchyma Sonography
CAD	Computer Aided Detection
CNN	Convolutional Neural Networks
DL	Deep Learning
DSC	Dice Coefficient
MRI	Magnetic Resonance Imaging
MSA	Multiple System Atrophy
PD	Parkinson’s Disease
PET	Positron Emission Tomography
SN	Substantia Nigra
SPECT	Single Photon Emission Computer Tomography
TCS	Transcranial Sonography
US	Ultrasound
